# Enzastaurin has anti-tumour effects in lung cancers with overexpressed JAK pathway molecules

**DOI:** 10.1038/bjc.2012.7

**Published:** 2012-02-14

**Authors:** T Shimokawa, M Seike, C Soeno, H Uesaka, A Miyanaga, H Mizutani, K Kitamura, Y Minegishi, R Noro, T Okano, A Yoshimura, A Gemma

**Affiliations:** 1 Department of Internal Medicine, Division of Pulmonary Medicine/Infection and Oncology, Nippon Medical School, 1-1-5, Sendagi, Bunkyo-ku, Tokyo 113-8603, Japan; 2 MediBIC, Tokyo, Japan

**Keywords:** lung cancer, enzautaurin, PKC inhibitor, gene expression, drug sensitivity

## Abstract

**Background:**

Enzastaurin, an oral serine–threonine kinase inhibitor, was initially developed as an ATP-competitive selective inhibitor against protein kinase C*β*. However, the mechanism by which enzastaurin contributes to tumourigenesis remains unclear.

**Methods:**

We analysed the anti-tumour effects of enzastaurin in 22 lung cancer cell lines to ascertain the potential for enzastaurin-based treatment of lung cancer. To identify molecules or signalling pathways associated with this sensitivity, we conducted a gene, receptor tyrosine kinases phosphorylation and microRNA expression profiling study on the same set of cell lines.

**Results:**

We identified eight genes by pathway analysis of molecules having gene-drug sensitivity correlation, and used them to build a support vector machine algorithm model by which sensitive cell lines were distinguished from resistant cell lines. Pathway analysis revealed that the JAK/STAT signalling pathway was one of the main ones involved in sensitivity to enzastaurin. Overexpression of JAK1 was observed in the sensitive cells by western blotting. Simultaneous administration of enzastaurin and JAK inhibitor inhibited enzastaurin-induced cell growth-inhibitory effect. Furthermore, lentiviral-mediated JAK1-overexpressing cells were more sensitive to enzastaurin than control cells.

**Conclusion:**

Our results suggested that the JAK1 pathway may be used as a single predictive biomarker for enzastaurin treatment. The anti-tumour effect of enzastaurin should be evaluated in lung cancer with overexpressed JAK pathway molecules.

Non-small-cell lung cancer (NSCLC) patients are usually diagnosed with advanced disease, and their prognosis remains poor despite improvements in chemotherapies (Mountain, 1997; Schiller *et al*, 2002; Ohe *et al*, 2007; Jemal *et al*, 2009). Recently, molecular-targeted therapies have been developed for NSCLC treatment. For example, NSCLC patients with epidermal growth factor receptor (EGFR) mutations have shown a dramatic response to EGFR inhibitors such as gefitinib and erlotinib (Mok *et al*, 2009; Maemondo *et al*, 2010). However, there remain many other molecular abnormalities in lung cancer that are as yet unexplored (Salgia and Skarin, 1998).

The protein kinase C (PKC) family of serine–threonine protein kinases has been implicated in several important cellular functions including proliferation, motility, invasion and apoptosis (Livneh and Fishman, 1997). Among the PKC isoforms, PKC*β* is known to be an important mediator of vascular endothelial growth factor (VEGF) (Xia *et al*, 1996; Yoshiji *et al*, 1999), the most potent angiogenic factor found in various tumours. Increased invasion and proliferation in tumours have also been associated with PKC*β* (Zhang *et al*, 2004). Overexpression and increased activity of PKC*β* have been implicated in transformation and tumourigenesis in lung cancer (Barr *et al*, 1997; Lahn *et al*, 2006). In several human cancers, PKC*β* expression is linked to poor prognosis, most notably in B-cell lymphoma (Shipp *et al*, 2002; Li *et al*, 2007). Biochemical analysis demonstrated that PKC*β* could target the phoshatidylinositol 3-kinase (PI3K)/AKT pathway and other signal transduction pathways (Graff *et al*, 2005; Rascoe *et al*, 2005). However, the mechanism by which PKC*β* contributes to tumourigenesis remains unclear.

The PKC*β* inhibitor enzastaurin, an oral serine–threonine kinase inhibitor, was initially developed as an ATP-competitive selective inhibitor against PKC*β* (Faul *et al*, 2003). Enzastaurin is now being evaluated in several phase II studies across a variety of more common tumour types including: breast, ovarian colon and prostate cancers (Mina *et al*, 2009; Vergote *et al*, 2009; Dreicer *et al*, 2010; Glimelius *et al*, 2010). It has also been evaluated as second- or third-line therapy for NSCLC in a phase II study (Oh *et al*, 2008; Chiappori *et al*, 2010). *In vitro*, sequence-dependent, synergistic anti-proliferative and proapoptotic effects of the combination of cytotoxic drugs and enzastaurin have been found in NSCLC cells (Rademaker-Lakhai *et al*, 2007; Morgillo *et al*, 2008; Tekle *et al*, 2008). These studies suggest that enzastaurin may have an activity against lung cancer.

In this study, we analysed the anti-tumour effects of enzastaurin in a panel of 22 lung cancer cell lines to ascertain the potential for enzastaurin-based treatment of lung cancer. We also conducted gene, receptor tyrosine kinases (RTKs) phosphorylation and microRNA (miRNA) profiling on the same set of cell lines to identify the molecules associated with sensitivity of lung cancer to enzastaurin treatment. The correlation between the cytotoxic activity of enzastaurin and the corresponding gene, RTKs phosphorylation and miRNA expression patterns has been examined to clarify the responsible mechanisms of the signalling pathway involved in the response of lung cancers to enzastaurin treatment.

## Materials and methods

### Cell lines

We used 22 lung cancer cell lines: A549, PC3, PC7, PC9, PC14, LC2/ad, ABC-1, RERF-LC-KJ, RERF-LC-MS, RERF-LC-AI adenocarcinoma (AC) cell lines and PC1, PC10, LK2, SQ5, QG56, EBC-1, LC1/sq squamous-cell carcinoma (SCC) cell lines and NCI-H69, NCI-N231, Lu135, SBC3, MS-1 small-cell lung carcinoma (SCLC) cell lines for this study. In addition, five cell lines comprising H1650, H1975, LC-1F, RERF-LC-OK and VMRC-LCD, were used as the test set for a validation study. A549, NCI-H69, NCI-N231, H1650 and H1975 were purchased from the American Type Culture Collection (ATCC, Manassas, VA, USA); RERF-LC-KJ, RERF-LC-AI, RERF-LC-OK, LC2-ad, SQ5, LC2/Ad, LC1/Sq, LC-1F and MS-1 were obtained from the RIKEN Cell Bank (Ibaraki, Japan) and PC1, PC3, PC7, PC9, PC10 and PC14 were obtained from Immuno-Biological Laboratories (Gunma, Japan); RERF-LC-MS, ABC-1, EBC-1, LK2, QG56 and VMRC-LCD were purchased from Health Science Research Resources Bank (Osaka, Japan). Lung cancer cell lines were maintained in RPMI 1640 medium (GIBCO, Carlsbad, CA, USA) supplemented with 10% fetal bovine serum.

### Drugs and growth-inhibition assay

Enzastaurin was kindly provided by Ely Lilly. Growth inhibition was assessed by MTS assay to examine the effect of enzastaurin on lung cancer cell lines. Cell suspensions (5000 cells per well) were seeded into 96-well plates and increasing concentrations of enzastaurin (0, 0.01, 0.1, 1.0, 10 and 100 *μ*M) were added. After incubation for 72 h at 37 °C, MTS was added to each well and incubated for 2 h at 37 °C, after which absorbance was measured using a microplate reader with a test wavelength of 450 nm. The IC_50_ value was defined as the concentration needed for 50% reduction of the growth by treatment with enzastaurin.

JAK inhibitor (JAK inhibitor I, Cat. No 420099) was purchased from Calbiochem (San Diego, CA, USA). A549 and RERF-LC-KJ cells (5000 cells per well) were seeded into 96-well plates. After 24 h, the cells were incubated for 72 h in the various concentrations of enzastaurin (0, 0.01. 0.1, 1.0, 10 and 100 *μ*M), with or without low-dose (1 *μ*M) JAK inhibitor.

### RNA isolation, cDNA array, RTKs phosphorylation antibody array and miRNA array

Total RNA was isolated from lung cancer cell lines with the use of TRIzol reagent (Invitrogen, Carlsbad, CA, USA), according to the manufacturer's instructions. High-density oligonucleotide array analysis was carried out using Affymetrix HG-U133A (22 282 probe sets) expression array, as previously described (Gemma *et al*, 2006). Scanning was performed with the GeneChip Scanner 3000 (Affymetrix, Santa Clara, CA, USA), and GeneChip analysis was based on the Affymetrix GeneChip Manual with GeneChip Operating Software version 1.0 (Affymetrix), and Microarray Database software. We also performed human RTKs phosphorylation antibody array, including 71 antibodies (RayBiotech, Inc., Norcross, GA, USA). MicroRNA expression profiles were analysed by TaqMan MicroRNA Array set version 2.0 containing 667 miRNAs and validated by TaqMan MicroRNA assay (Applied Biosystems, Foster City, CA, USA).

### Western blot analysis

Cells were lysed in buffer containing 50 mM Tris–HCl, pH 7.6, 150 mM NaCl, 0.1% sodium dodecyl sulphate, 1% Nonidet P-40 and 0.5% sodium deoxycholate. The lysates were kept on ice for 30 min, and then centrifuged at 13 000 **g** for 30 min. The supernatant was collected and 10 *μ*g of protein were separated by gel electrophoresis on 10% gels, transferred to nitrocellulose membranes and detected by immunoblotting using a chemiluminescence system (GE Healthcare Bio-Sciences Corp., Piscataway, NJ, USA). The antibodies detecting JAK1, STAT3, phospho-STAT3 (p-STAT) and *β*-actin were purchased from Cell Signaling Technology (Beverley, MA, USA).

### Lentiviral-mediated JAK1-overexpressing cells

Expression plasmid vector pEZ-Lv151 was used for lentiviral vector production (GeneCopoeia, Rockville, MD, USA). The coding sequence of human JAK1 or enhanced green fluorescent protein (EGFP) was inserted under the transcriptional control of the CMV promoter in pEZ-Lv151. The human JAK1 lentiviral expression plasmid (Ex-T8644-Lv151) or EGFP plasmid (Ex-EGFP-Lv151) was cotransfected into 293Ta cells with the Lenti-Pac HIV Packaging Mix (GeneCopoeia). Lentivirus-containing supernatants were harvested 48 h after transfection. The lentivirus particles were purified and stored at −80 °C in aliquots until use.

To establish stable JAK1-overexpressing cell lines, A549 cells were transduced with serial dilutions of lentiviral supernatant in the presence of 5 *μ*g ml^–1^ polybrene and selected by 0.8 ng ml^–1^ geniticine. After antibiotic selection for 3 weeks, stable overexpressing JAK1 cells (LV-JAK1 A549 cells) were obtained.

### Statistical analyses

Data analysis for the correlation coefficients that revealed the correlation between the drug activity patterns and the gene expression patterns was principally done by a modified National Cancer Institute programme (Miyanaga *et al*, 2008). We used pathway analysis to provide a viewpoint of the biological function of genes within the proposed classifier. Pathway analysis was done using the Pathway Architect software (Stratagene, La Jolla, CA, USA). The pathways showing the relationships among the genes on the list were drawn by selecting all molecules on the pathway edit window. All relationships among the molecules were retrieved from the database, with this information being derived from PubMed abstracts by natural language processing technology. The function was done by selecting the data of maximum reliability (MAX) by choosing all modes of interactions including ‘Promoter Binding’, ‘Regulation’, ‘Protein Modification’ and ‘Expression’ and by taking the relationships supported by three or more consistent data sources. Next, we picked out the incorporated genes from the imported gene list used at the onset of the pathway analysis, except the subunits of the target gene. Thus, a list of the genes associated with drug response was established with respect to not only gene expression profile data but also the biological functions of altered/associated genes. Data from the listed genes were used to build a support vector machine (SVM) model with ArrayAssist software (Stratagene) to predict the drug response (IC_50_). The SVM algorithm model with Gaussian kernels was used to distinguish sensitive cells from resistant cells, using biomarkers identified by the gene expression-enzastaurin drug sensitivity correlation and pathway analysis. The classification ability of the genes was evaluated using leave-one-out cross-validation.

## Results

### Effect of enzastaurin on the growth of lung cancer cells

Growth-inhibitory effects of enzastaurin on lung cancer cell lines were assessed by MTS assay.

**Figure 1:**
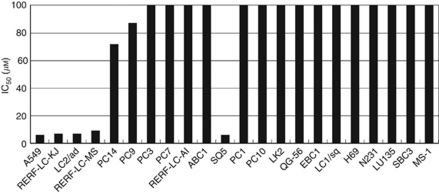
IC_50_ values for 22 lung cancer cell lines responding to enzastaurin treatment by MTS assay. According to sensitivity to enzastaurin, these 22 cell lines were classified as sensitive (IC_50_ of ⩽10 *μ*M) or resistant (IC_50_ of >50 *μ*M).

Figure 1 shows the sensitivity to enzastaurin among the 22 lung cancer cells. Based on the IC_50_, the 22 cell lines were classified into two groups, namely: enzastaurin sensitive and enzastaurin resistant. Five cell lines (A549, RERF-LC-KJ, LC2/ad, RERF-LC-MS and SQ5) were sensitive (IC_50_ of ⩽10 *μ*M), and the remaining 17 cell lines were resistant to enzastaurin (IC_50_ of >50 *μ*M). The five cell lines sensitive to enzastaurin consisted of four AC (4/10, 40%) and one SCC (1/7, 14%) cell line; no SCLC (0/5) cell lines were enzastaurin sensitive. These results suggest that enzastaurin has anti-tumour activity against NSCLC.

### Gene expression-drug sensitivity correlation

**Figure 2:**
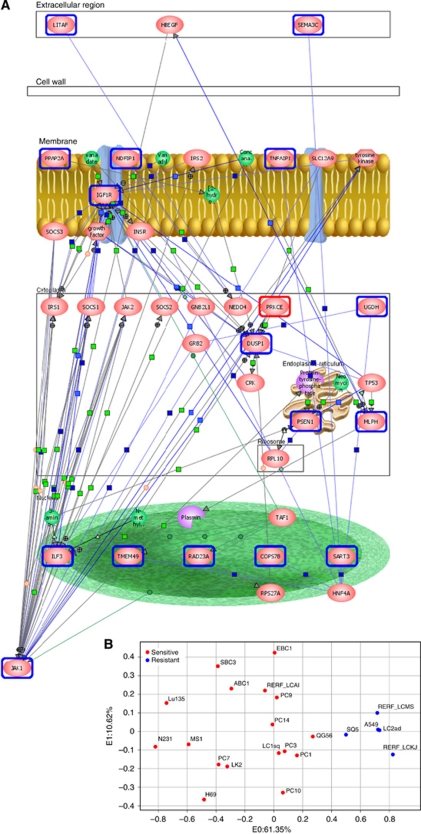
Sixteen genes associated with enzastaurin response were established by pathway analyses and prediction of drug response using an eight-gene signature. (**A**) Sixteen genes (blue circle) associated with enzastaurin response and PKC (red circle) belonged to the same signal pathway. (**B**) Principal component analysis based on the eight-gene profile correctly distinguished the sensitive cells from the resistant ones. The colour reproduction of this figure is available at the *British Journal of Cancer* online.

We have previously performed gene expression profile analysis of the same set of 22 lung cell lines by Affymetrix GeneChip (Gemma *et al*, 2006). First, we used the MTS results for enzastaurin for the development of a molecular model of sensitivity to enzastaurin. Twenty-three genes were significantly correlated with sensitivity to enzastaurin (correlation coefficients of >0.65). Next, pathway analysis was performed using the 23 genes to provide a viewpoint of the biological function of the genes, as previously described (Miyanaga *et al*, 2008). Pathway analysis removed the incorporated genes out of the imported 23 genes. Sixteen genes, associated with sensitivity to enzastaurin, were identified based on the biological functions of altered/associated genes (Table 1; Figure 2A). Pathway analysis revealed that JAK1 was the final target gene for the sensitivity to enzastaurin in lung cancer cells (Figure 2A). We next identified the optimal number of genes whose expression could accurately distinguish the sensitive cells from the resistant ones. Analysis of variance (ANOVA) was done to remove the genes with variance. The top eight genes (DUSP1, ILF3, LITAF, JAK1, COPS7B, RAD23A, TNFAIP1 and MIRN21/TMEM49) according to the ANOVA were subsequently found to be the minimum number necessary for prediction of drug response (Figure 2B; Table 1). We used the eight most strongly correlated genes to build an SVM algorithm model by which the five sensitive cells were distinguished from the 17 resistant cells. Overall, the SVM classification based on the above-mentioned eight genes, correctly classified the sensitivity to enzastaurin of all of the 22 cells (data not shown). Next, we examined the robustness of the eight-gene predictor, for classifying cells into the enzastaurin-sensitive group, in an independent set of NSCLC cells, and found that the eight-gene predictor correctly classified all five resistant cells (Table 2). Thus, we had ultimately identified an eight-gene signature that was validated for its ability to predict the sensitivity to enzastaurin in an independent set of lung cancer cells.

### RTKs phosphorylation and miRNA expression-drug sensitivity correlation

**Figure 3:**
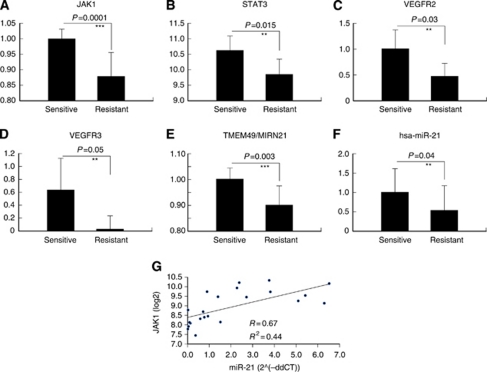
JAK1, VEGFR2, VEGFR3 and miR-21 were correlated with drug response. (**A** and **B**) JAK1 and STAT3 gene expression levels were significantly higher in the sensitive cell group than in the resistant cell group. (**C** and **D**) Elevated levels of VEGFR2 and VEGFR3 expression were observed in sensitive cells. (**E**) Expression of MIRN21/TMEM49 was significantly higher in sensitive cells than in resistant cells, by gene-chip analysis. (**F**) Mature miR-21 expression was significantly higher in sensitive cells than in resistant cells by quantitative RT–PCR analysis. (**G**) Quantitative comparison of miR-21 and JAK1 showed a significant positive correlation between these two molecules. ^**^P<0.05 when compared with the resistant cells. ^***^P<0.01 when compared with the resistant cells.

Pathway analysis revealed that JAK1 was an important gene for the sensitivity to enzastaurin in lung cancer cells. JAK1 and its downstream STAT3 gene expression levels of sensitive cells were significantly higher than those of resistant cells (Figures 3A and B). To further clarify the signalling mechanism correlated with the sensitivity to enzastaurin, we also examined RTKs phosphorylation expression profiles of the same set of 22 lung cancer cells. The top 10 RTKs phosphorylation associated with enzastaurin sensitivity are shown in Table 3 (correlation coefficients of >0.50). Pathway analysis using the 23 genes and 10 RTKs phosphorylation associated with sensitivity to enzastaurin also revealed that JAK/STAT signal pathway was mainly involved in the drug response (data not shown). Among the 10 RTKs phosphorylation, the expression of two RTKs mainly associated with angiogenesis and lymphangiogenesis (VEGFR2 and VEGFR3) was significantly elevated in sensitive cells compared with in resistant cells (Figures 3C and D).

In order to investigate post-transcriptional regulation, miRNA microarray analysis of the 22 cells was also performed. We identified 13 miRNAs correlated with enzastaurin sensitivity (correlation coefficients of >0.50) (Table 3). Interestingly, MIRN21/TMEM49, a host gene of miR-21, was included among the eight genes associated with enzastaurin sensitivity, and was expressed at significantly higher levels in sensitive cells compared with in resistant cells (Figure 3E). In addition, a correlation between miR-21 and enzastaurin sensitivity was found in miRNA array analysis (correlation coefficients −0.53) (Table 3). Recent reports demonstrated that miR-21 is a major miRNA that may play an oncogenic role in lung carcinogenesis (Volinia *et al*, 2006; Yanaihara *et al*, 2006; Seike *et al*, 2009). The expression levels of miR-21 were examined by real-time quantitative RT–PCR. miR-21 expression was significantly higher in sensitive cells than in resistant cells (*P*<0.05, paired *t*-test) (Figure 3F). The quantitative comparison of miR-21 and JAK1 showed a significant positive correlation between these two (Pearson's correlation, *r*=0.67, *P*<0.05) (Figure 3G). We ultimately recognised JAK1, VEGFR2, VEGFR3 and miR-21 as factors concerned with sensitivity to enzastaurin. In particular, JAK1 is the most significant molecule involved in drug response.

### JAK1 expression effect on drug sensitivity in A549 cells

**Figure 4:**
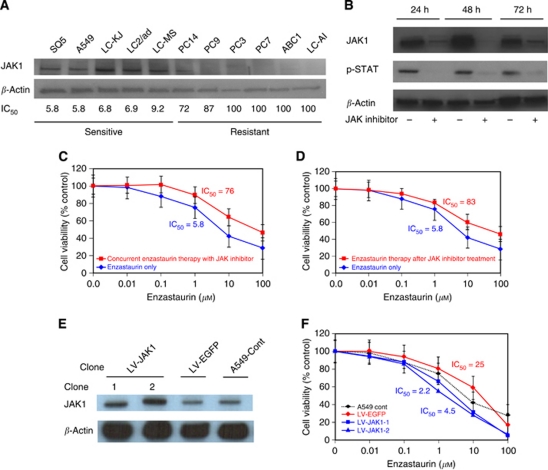
Effect of combination therapy with enzastaurin and JAK1 expression on cell growth in lung cancer cells. (**A**) JAK1 expression levels were significantly higher in the sensitive cell group than in the resistant cell group, by western blotting. (**B**) Completed inhibition of JAK1/STAT signalling by JAK1 inhibitor in A549 cells. P-STAT3 was completely inhibited until 72 h after the treatment of 1 *μ*M JAK inhibitor. (**C**) Enzastaurin treatment with JAK inhibitor for 72 h was examined in A549 cells. Each result is expressed as cell viability in treated samples compared with the untreated sample (100%) for enzastaurin alone and concurrent therapy with the 1 *μ*M JAK inhibitor treatment. (**D**) The effect of JAK inhibitor treatment (1 *μ*M) for 24 h followed by enzastaurin treatment for 72 h was examined in A549 cells. (**E**) Lentiviral-mediated production of JAK1 in A549 cells. Western blotting showed that JAK1 expression levels were significantly higher in two LV-JAK1 clones than in the control clones. (**F**) Enzastaurin treatment for 72 h was examined in LV-JAK1-A549 cells. Each result is expressed as cell viability in the treated samples compared with the untreated sample (100%) for enzastaurin therapy.

To investigate further the effect of JAK1 on sensitivity to enzastaurin, JAK1 protein expression of 11 NSCLC cells was evaluated by western blot analysis. Elevated JAK1 protein was observed in enzastaurin-sensitive NSCLC cells (Figure 4A). Next, we inhibited JAK1 protein using JAK1 inhibitor in enzastaurin-sensitive A549 and RERF-LC-KJ cells. After the treatment of JAK inhibitor (1 *μ*M), JAK1 and its downstream p-STAT3 expression was completely diminished until 72 h in A549 cells (Figure 4B). We examined the effect of enzastaurin and JAK inhibitor combination therapy on cell growth. Concurrent JAK inhibitor and enzastaurin therapy significantly decreased the growth-inhibitory effect of enzastaurin, compared with enzastaurin monotherapy in enzastaurin-sensitive A549 cells (Figure 4C). Enzastaurin therapy after JAK inhibitor 1 *μ*M treatment also diminished the growth-inhibitory effect of enzastaurin, compared with enzastaurin monotherapy in A549 cells (Figure 4D). The IC_50_ values of concurrent enzastaurin with JAK inhibitor and enzastaurin therapy after JAK inhibitor were 76 and 83, respectively, whereas that of enzastaurin monotherapy was 5.8 (Figures 4C and D). In addition, RERF-LC-KJ cells, which are also sensitive to enzastaurin, showed resistance after JAK inhibitor therapy in combination with enzastaurin (data not shown). In RERF-LC-KJ cells, both IC_50_ values of concurrent enzastaurin with JAK inhibitor and enzastaurin therapy after JAK inhibitor were over 100, whereas that of enzastaurin monotherapy was 6.8. To confirm further the ability of JAK1 to indicate drug sensitivity to enzastaurin, we developed a lentiviral vector for the expression of JAK1 and established stable JAK1-overexpressing A549 cells (LV-JAK1-A549 cells). Western blot analysis showed the overexpression of JAK1 in LV-JAK1-A549 cells (Figure 4E). The growth-inhibitory effect of enzastaurin on LV-JAK1-A549 cells was assessed by MTS assay. The drug sensitivities of two LV-JAK1-A549 cells were greater than those in the control cells (Figure 4F). The IC_50_ values of two LV-EGFP A549 cells were 2.2 and 4.5, respectively, whereas that of LV-EGFP A549 cells was 25 (Figure 4F). These results indicate that JAK1 expression contributed to the drug sensitivity and could be used as a drug-sensitive marker to enzastaurin in lung cancer cells.

### JAK/STAT3 pathway directly activates miR-21

**Figure 5:**
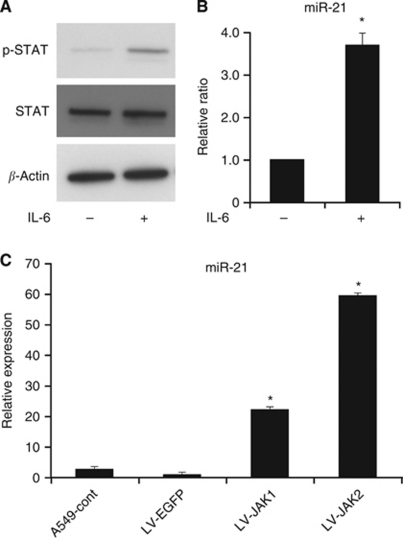
Association between JAK1 and miR-21 expression. (**A**) p-STAT3 was overexpressed after IL-6 stimulation of A549 cells for 24 h. (**B**) After IL-6 stimulation, miR-21 expression was significantly increased, as measured by qRT–PCR analysis. (**C**) MiR-21 expression of two LV-JAK1 cells was significantly higher than in the control cells, as measured by qRT–PCR analysis. Data were mean±s.d. from three independent experiments. ^*^P<0.05 when compared with the respective parent cells.

A significant correlation between JAK1 and miR-21 was found in our set of NSCLC cells (Figure 3G). STAT3 is a transcription factor activated by JAK1, and its binding to the target sites in miR-21 promoter upon IL-6 induction has been reported previously (Löffler *et al*, 2007; Iliopoulos *et al*, 2010). To verify the association between JAK1 and miR-21, miR-21 expression was quantified after the stimulation of IL-6 by qRT–PCR analysis. Upon IL-6 exposure, p-STAT3 expression was significantly upregulated, resulting in the overexpression of miR-21 at 24 h in A549 cells (Figures 5A and B). We also evaluated the miR-21 expression in LV-LAK1 A549 cells. In the JAK1-overexpressing cells, miR-21 expression was significantly higher than in parent cells (Figure 5C). These results supported the concept that miR-21 is directly induced by JAK/STAT signalling in NSCLC cells.

## Discussion

Enzastaurin has recently been evaluated as second- or third-line therapy of NSCLC in a phase II study (Oh *et al*, 2008; Chiappori *et al*, 2010). Synergistic effects of the combination of enzastaurin and cytotoxic drugs including cisplatin, gemcitabine and pemetrexed have been found in NSCLC cells in an *in vitro* study (Rademaker-Lakhai *et al*, 2007; Morgillo *et al*, 2008; Tekle *et al*, 2008). A recent study showed that enzastaurin inhibited *in vivo* metastasis of NSCLC cells (Körner *et al*, 2010). It is known that PKCs mediate the regulation of the cell cycle; enzastaurin is also able to inhibit several proteins involved in cell-cycle regulation, for example, E2F-1 associated with G1/S checkpoint and Cdc25C resulting in G2/M checkpoint (Tekle *et al*, 2008). These checkpoint arrests provide the tumour cells with the opportunity to repair their DNA, which has been damaged by cytotoxic drugs. Reduction of E2F-1 expression and phosphorylated Cdc25C by enzastaurin might explain the abrogation of the checkpoint arrest and could facilitate cytotoxic drug-damaged cells to undergo apoptosis. Furthermore, a recent study demonstrated that enzastaurin had a cooperative effect with gefitinib and was able to revert gefitinib resistance in cancer cells through the inhibition of Akt and VEGF pathways (Gelardi *et al*, 2008). These studies suggest that enzastaurin might be a promising novel agent in NSCLC patients.

Enzastaurin inhibited the downstream PKC*β* signalling, PI3K/AKT pathway and the phosphorylation of glycogen synthase kinase-3*β* (Keyes *et al*, 2002; Graff *et al*, 2005). Anti-tumour and anti-angiogenic activity of enzastaurin was also demonstrated in tumour xenograft models, including NSCLC, and was confirmed using a standardised clonogenic assay in patient-derived tumour explants (Keyes *et al*, 2004). Significant reduction of VEGF protein levels following enzastaurin treatment, together with a significant decrease in intratumoural vessel density, has been demonstrated *in vivo* (Keyes *et al*, 2004). In the current study using a RTKs phosphorylation antibody array, we found elevated levels of VEGFR2 and VEGFR3 in the enzastaurin-sensitive cells. Our results are in agreement with previous data concerning enzastaurin and anti-angiogenic activity. These findings demonstrated that lung cancer cases with activated angiogenic activity should respond to enzastaurin treatment.

In this study, using gene-chip and pathway analysis, we identified 16 genes that correlated with sensitivity to enzastaurin. Pathway analysis also revealed that JAK1 was the most important molecule affected by enzastaurin treatment of NSCLC. The JAK is a non-RTK and can activate STAT3 transcriptional factor. The STAT3 is also persistently activated in about half of NSCLC tumours and is involved in tumour invasion, metastasis and angiogenesis through differential gene regulation (Haura *et al*, 2005; Song *et al*, 2011). Increased levels of JAK1 and STAT3 were observed in the sensitive cells in this study. Knockdown of JAK resulting in p-STAT3 also diminished the growth-inhibitory effect of enzastaurin in the sensitive cells. In contrast, overexpression of JAK1 by lentiviral-mediated production enhanced the drug sensitivity to enzastaurin in the sensitive cells. These results suggest that JAK expression levels can be used as predictive markers of enzastaurin sensitivity. Non-small-cell lung cancer patients with an activated JAK/STAT3 pathway are suitable cases for enzastaurin treatment.

MicroRNAs are small non-coding RNA molecules of about 20 nucleotides that are frequently located at chromosomal regions deleted or amplified in cancers, suggesting that miRNAs are a new class of genes involved in human tumourigenesis (Lu *et al*, 2005; Volinia *et al*, 2006; Yanaihara *et al*, 2006; Seike *et al*, 2009). Recently, miRNAs have been demonstrated as diagnostic and prognostic markers in lung cancer (Yanaihara *et al*, 2006; Seike *et al*, 2009). We previously reported that the inhibition of miR-21, whose upregulation is associated with EGFR mutations, can be a therapeutic strategy, either as a monotherapy or in combination with EGFR-TKI treatment (Seike *et al*, 2009). In this study, expression of miR-21 and its host gene, TMEM49, were significantly higher in enzastaurin-sensitive cells than in enzastaurin-resistant cells. In addition, a significant positive correlation was observed between miR-21 and JAK1. The STAT3 reportedly signals IL-6-induced upregulation of miR-21 in multiple myeloma cells (Löffler *et al*, 2007). We confirmed that JAK1 and its downstream target STAT3, containing three binding sites of miR-21 promoter, directly activated miR-21 in NSCLC cells. These results suggest that, in lung cancer, miR-21 affects the response to enzastaurin through the JAK/STAT signalling pathway.

In conclusion, we have identified unique molecules; genes, RTKs and miRNAs that are correlated with sensitivity to enzastaurin and have constructed an eight-gene signature to distinguish the sensitive cells from the resistant cells. Furthermore, we demonstrate that JAK1 is the most significant factor concerned in response to enzastaurin. Patient selection based on the JAK expression might be useful for future clinical development of enzastaurin therapy in NSCLC.

## Figures and Tables

**Table 1:** Unique genes correlated with sensitivity to enzastaurin

**Gene symbol**	**Gene title**	**F-statistic**	***P*-value**	**Correlation coefficients**	**Eight-gene predictor**
DUSP1	Dual specificity phosphatase 1	49.2	8.39E–07	−0.69	^*^
ILF3	Interleukin enhancer binding factor 3, 90 kDa	48.5	1.10E–06	0.67	^*^
LITAF	Lipopolysaccharide-induced TNF factor	36.0	9.75E–06	−0.70	^*^
JAK1	Janus kinase 1 (a protein tyrosine kinase)	27.1	6.36E–05	−0.65	^*^
COPS7B	COP9 constitutive photomorphogenic homologue subunit 7B (Arabidopsis)	19.3	5.48E–04	0.66	^*^
RAD23A	RAD23 homologue A (*S. cerevisiae*)	23.0	9.10E–04	0.74	^*^
TNFAIP1	Tumour necrosis factor, *α*-induced protein 1 (endothelial)	19.5	0.002	−0.65	^*^
MIRN21///TMEM49	Transmembrane protein 49///microRNA 21	14.1	0.003	−0.66	^*^
PSEN1	Presenilin 1 (Alzheimer disease 3)	9.5	0.012	−0.65	
PPAP2A	Phosphatidic acid phosphatase type 2A	11.3	0.014	−0.75	
IGF1R	Insulin-like growth factor 1 receptor	10.6	0.019	−0.66	
SART3	Squamous cell carcinoma antigen recognised by T cells 3	9.4	0.019	0.65	
NDFIP1	Nedd4 family interacting protein 1	6.0	0.029	−0.66	
MLPH	Melanophilin	8.2	0.034	−0.65	
SEMA3C	Sema domain, immunoglobulin domain (Ig), short basic domain, secreted (semaphorin) 3C	5.9	0.056	−0.67	
UGDH	UDP-glucose dehydrogenase	5.9	0.062	−0.68	

Abbreviations: ANOVA=analysis of variance; TNF=tumour necrosis factor.

Note: F-statistic and *P*-values were calculated by ANOVA. ^*^Genes used as eight-gene predictor are shown.

**Table 2:** Validation of the eight-gene predictor by examining the SVM value in an independent set of five NSCLC cell lines

		**Histology**	**IC_50_ (*μ*M)**	Predicted class*
1	H1650	AC	>100	Resistant
2	H1975	AC	>100	Resistant
3	RERF-LC-OK	AC	>100	Resistant
4	VMRC-LCD	AC	>100	Resistant
5	LC-1F	SCC	>100	Resistant

Abbreviations: AC=adenocarcinoma; SVM=support vector machine; NSCLC=non-small-cell lung cancer.

Note: ^*^Cell lines were classified as sensitive (IC_50_ of ⩽10 *μ*M) and resistant (IC_50_ of >50 *μ*M) to enzastaurin.

**Table 3:** Kinase and miRNA correlated with the sensitivity to enzastaurin

	**Kinase**	**F-statistic**	***P*-value**	**Correlation coefficients**
*(a)*				
1	M-CSFR	11.51	0.02	−0.82
2	VEGFR2	9.17	0.03	−0.68
3	FER	9.00	0.02	−0.60
4	EphA1	7.58	0.02	−0.61
5	VEGFR3	6.76	0.05	−0.58
6	TNK1	4.45	0.09	−0.71
7	NGFR	3.73	0.11	−0.68
8	MATK	2.95	0.15	−0.52
9	Hck	2.26	0.20	−0.53
10	SYK	1.82	0.23	−0.58
				
	**miRNA**	**F-statistic**	***P*-value**	**Correlation coefficients**
*(b)*				
1	hsa-miR-15a^*^	18.56	0.0004	0.51
2	hsa-miR-454^*^	16.65	0.0006	0.53
3	hsa-miR-92a	15.96	0.0007	0.52
4	hsa-miR-301b	12.49	0.0021	0.54
5	hsa-miR-130b	11.85	0.0026	0.54
6	hsa-miR-106b^*^	11.42	0.0032	0.52
7	hsa-miR-345	9.25	0.01	0.54
8	hsa-miR-31	7.25	0.05	−0.76
9	hsa-let-7a	4.04	0.09	0.54
10	hsa-miR-193b	2.76	0.14	−0.64
11	hsa-miR-193b^*^	2.76	0.15	−0.61
12	hsa-miR-21	2.24	0.18	−0.53
13	hsa-miR-30c-2^*^	1.93	0.24	−0.52

Abbreviations: ANOVA=analysis of variance; miRNA=microRNA.

Note: F-statistic and *P*-values were calculated by ANOVA. ^*^The miRNA name used in TaqMan microRNA array analysis.
